# New variants of COVID‐19 (XBB.1.5 and XBB.1.16, the “Arcturus”): A review of highly questioned concerns, a brief comparison between different peaks in the COVID‐19 pandemic, with a focused systematic review on expert recommendations for prevention, vaccination, and treatment measures in the general population and at‐risk groups

**DOI:** 10.1002/iid3.1323

**Published:** 2024-06-27

**Authors:** Homa Pourriyahi, Nima Hajizadeh, Mina Khosravi, Homayoun Pourriahi, Sanaz Soleimani, Nastaran Sadat Hosseini, Arash Pour Mohammad, Azadeh Goodarzi

**Affiliations:** ^1^ Student Research Committee, School of Medicine Iran University of Medical Sciences Tehran Iran; ^2^ School of Medicine Iran University of Medical Sciences Tehran Iran; ^3^ Rasool Akram Medical Complex Clinical Research Development Center (RCRDC), School of Medicine Iran University of Medical Sciences Tehran Iran; ^4^ School of Medicine Isfahan University of Medical Sciences Isfahan Iran; ^5^ Department of Dermatology, Rasool Akram Medical Complex Clinical Research Development Center (RCRDC), School of Medicine Iran University of Medical Sciences Tehran Iran

**Keywords:** BA.5, Cilgavimab, consensus opinion, COVID‐19, guidelines, Nirmatrelvir, Omicron, pre‐exposure prophylaxis, recommendations, Ritonavir, SARS‐CoV‐2, Tixagevimab, XBB.1.16, XBB.1.5

## Abstract

**Introduction:**

The COVID‐19 pandemic has taken many forms and continues to evolve, now around the Omicron wave, raising concerns over the globe. With COVID‐19 being declared no longer a “public health emergency of international concern (PHEIC),” the COVID pandemic is still far from over, as new Omicron subvariants of interest and concern have risen since January of 2023. Mainly with the XBB.1.5 and XBB.1.16 subvariants, the pandemic is still very much “alive” and “breathing.”

**Methods:**

This review consists of five highly concerning questions about the current state of the COVID Omicron peak. We searched four main online databases to answer the first four questions. For the last one, we performed a systematic review of the literature, with keywords “Omicron,” “Guidelines,” and “Recommendations.”

**Results:**

A total of 31 articles were included. The main symptoms of the current Omicron wave include a characteristically high fever, coughing, conjunctivitis (with itching eyes), sore throat, runny nose, congestion, fatigue, body ache, and headache. The median incubation period of the symptoms is shorter than the previous peaks. Vaccination against COVID can still be considered effective for the new subvariants.

**Conclusion:**

Guidelines recommend continuation of personal protective measures, third and fourth dose boosters, along with administration of bivalent messenger RNA vaccine boosters. The consensus antiviral treatment is combination therapy using Nirmatrelvir and Ritonavir, and the consensus for pre‐exposure prophylaxis is Tixagevimab and Cilgavimab combination. We hope the present paper raises awareness for the continuing presence of COVID and ways to lower the risks, especially for at‐risk groups.

## Key points

- The COVID pandemic is far from over, with new variants of interest and concern rising since January of 2023 (mainly XBB.1.5 and XBB.1.16 subvariants).
- The main symptoms of the current Omicron wave include a high fever, coughing, conjunctivitis (with itching of the eyes), sore throat, runny nose and congestion, fatigue, body ache, and headache.
- The median incubation period for the current peak was calculated to be 3.8 days which is shorter than the incubation period calculated for most of the previous peaks; and the median duration of the symptoms was calculated to be 5 days for the current peak which is shorter than the duration calculated for the previous peaks by 3–5 days.
- A cumulative 64.42% of the population worldwide are fully vaccinated against COVID‐19 as of June 16, 2023. Vaccination against COVID can still be considered mostly effective against new symptoms, severe disease, long COVID, and death for the new subvariants.
- Guidelines recommend continuation of personal protective measures, third and fourth dose boosters, particularly in individuals above 60, immunocompromised patients, and healthcare personnel, along with administration of bivalent messenger RNA vaccine boosters.
- The consensus option for antiviral treatment is combination therapy using Nirmatrelvir and Ritonavir, and the consensus for pre‐exposure prophylaxis is Tixagevimab and Cilgavimab combination.

## Graphical abstract

This article consists of five highly concerning questions asked about the current COVID peak. For the first four questions, which are on current variants of concern, a comparison of major COVID peaks, efficacy of vaccination in preventing severe COVID, long COVID, hospitalization, and mortality, and determination of high‐risk groups, we have gathered data in a narrative review style. For the fifth question, which is on the recommendations and expert opinions for social measures, vaccination, and treatment of COVID among the general population and those at risk, we performed a systematic review of the literature, according to Preferred Reporting Items for Systematic reviews and Meta‐Analyses (PRISMA).

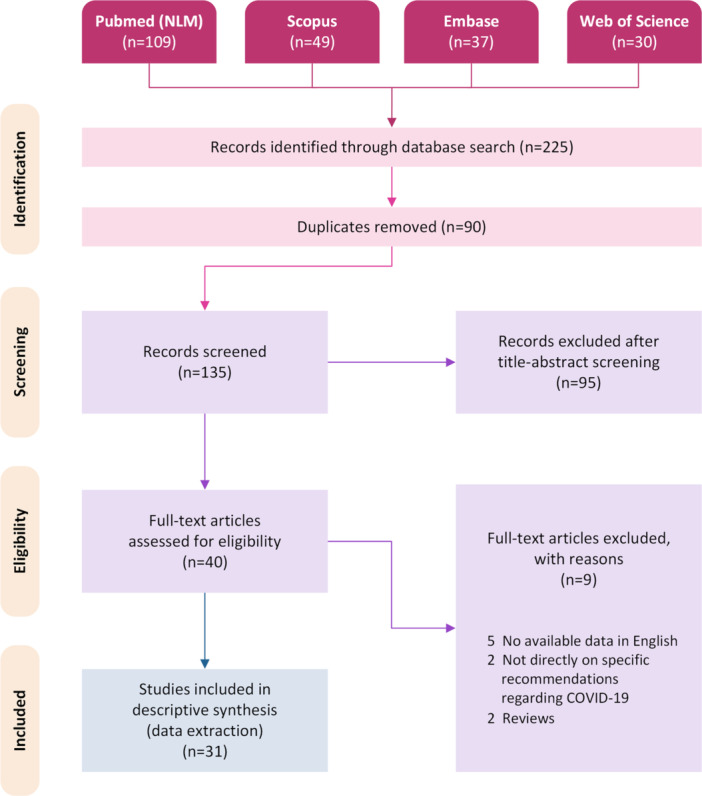

## 1 INTRODUCTION

The coronavirus disease 2019 (COVID‐19) pandemic, caused by the severe acute respiratory syndrome coronavirus 2 (SARS‐CoV‐2), has caused great proportions of mortality and morbidity around the world with over 766 million confirmed cases of COVID‐19 and over 6.9 million COVID‐caused deaths around the world reported by the WHO as of May 23, 2023.^1^ Nevertheless, the World Health Organization (WHO) claims that the infection rates are higher than what is reported in 2023, because of reduced numbers of tests performed and slowed‐down reporting.^1^

The five variants known to have contributed to the major COVID‐19 waves worldwide, namely, Alpha, Beta, Gamma, Delta, and the Omicron parent lineage (B.1.1.529), are all currently declared to be “previously circulating variants of concern,” according to the WHO.^2^

The Omicron parent lineage rose to concern in late 2021 and has been the dominant strain of SARS‐COV‐2 in many countries since then.^3^ Studies have shown that Omicron variant's transmissibility is very high,^3^ and owing to this higher transmission rate and an increased virulence compared to the previous types, it has continually caused significant hospitalization rates and has put a huge pressure on healthcare systems in a lot of countries.^4^ Omicron can potentially cause severe disease with noticeable morbidity and mortality, mostly evident in vulnerable populations,^5^ although the risk of severe disease and death after infection is lower than previous variants.^4^ During the Omicron peak, various areas showed high household transmissions due to asymptomatic as well as presymptomatic spread, in spite of the institution of firm nonpharmaceutical interventions.^6^ Moreover, current COVID‐19 vaccines and immunotherapies have been proven less effective for this variant, such that vaccinated subjects and patients who have recovered from COVID‐19 are liable to the disease.^5^ These findings suggest that proper timely management of SARS‐CoV‐2, including both its early detection and isolation, is crucial.^6^

With that being said, it is vital to know the latest face of the disease and update our knowledge of the current situation of the pandemic, as the COVID‐19 pandemic has taken many forms and continues to evolve, now around the Omicron wave, raising concerns over the globe.^5^ As of January 2023, new Omicron subvariants have been identified, with a rising number of cases, and their status being upgraded to “variant of interest,”^7^ and “variant of concern” by the WHO.^8^ Therefore, even though the pandemic was recently declared as no longer being a “public health emergency of international concern (PHEIC)” on May 5, 2023,^9^ and the end of the “Federal COVID‐19 Public Health Emergency (PHE)” Declaration as of May 11, 2023,^10^ it is still very much “alive” and “breathing.”

In this review, we will address the highly concerning questions regarding the current situation and will provide a systematic review of the latest guidelines and recommendations for prevention strategies, social measures, treatment options, and booster vaccinations, for both the general population and at‐risk groups. To reach this aim, we devised five important questions, namely:

1.1. How many COVID peaks has there been? How and when did the current peak start and how is it spreading in the world geographically? What are the incidence rates across countries? Which are the current peak's variants of interest (VOIs) and variants of concern (VOCs)?
1.2. What are the differences between the current peak and the previous ones (in terms of time of detection, country found, time of rise to concern, most common signs and symptoms, pathway of transmission, median incubation period, median duration of symptoms, long‐lasting symptoms (long COVID) cumulative percentage of world population fully vaccinated, hospitalization risk worldwide (hazard ratio), intensive care unit (ICU) admit percentage and adjusted hazard ratio, total deaths per 1 million in each time period)?
1.3. How effective has the previous vaccination been in preventing severe COVID, long COVID, and hospitalization, along with lowering death rates in the current peak?
1.4. Who are the high‐risk groups for transmission, being asymptomatic carriers, having severe disease or mortality in the current peak?
1.5. What are the recommendations and consensus opinions on the current peak? (for which we performed a systematic review).

## 2 METHODS

This article consists of five highly concerning questions asked about the current COVID peak. For the first four questions, we performed a narrative review with a comprehensive search on Pubmed (NLM), Scopus, Web of Science, and Embase, as well as the Internet for relevant news or online databases, and gathered data in a narrative review style. For the fifth question, which is on the recommendations and expert opinions for social measures, vaccination, and treatment of COVID among the general population and those at risk, we performed a systematic review of the literature; for which the details are presented below.

### 2.1 Protocol and registration

We have followed the Preferred Reporting Items for Systematic Reviews and Meta‐Analyses (PRISMA) guidelines, to conduct, prepare the manuscript and report this systematic review.^11^ This systematic review was not registered.

### 2.2 Search strategy

**Figure 1:**
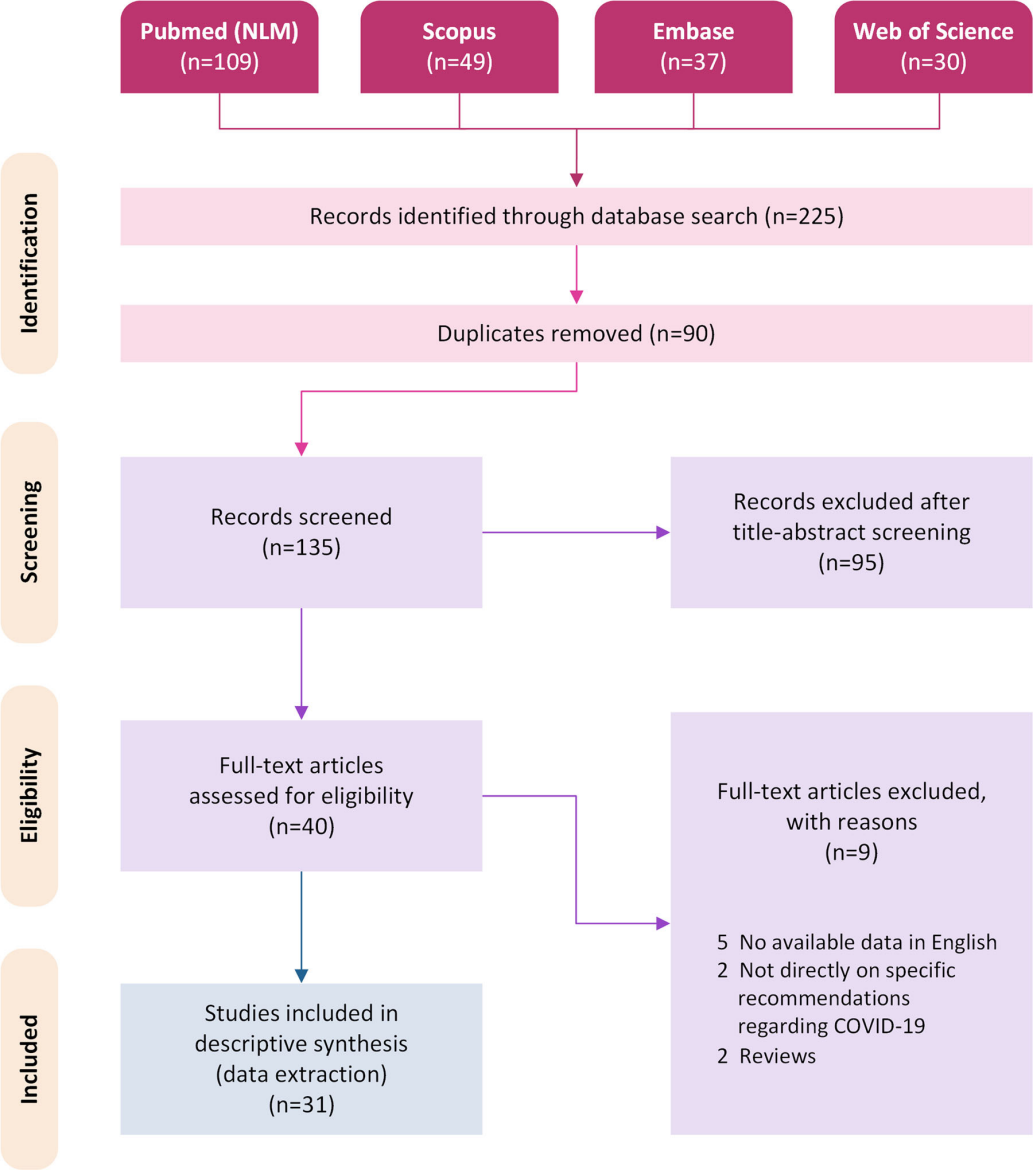
PRISMA flow diagram. PRISMA, Preferred Reporting Items for Systematic reviews and Meta‐Analyses.

We performed a comprehensive electronic search through the international databases, namely, PubMed (NLM), Scopus, Web of Science, and Embase with a starting point of January 1, 2023, up until March 31, 2023, and all articles regarding the recommendations and expert opinions for social measures, vaccination and treatment of COVID among the general population and those at risk, were initially retrieved using the names of the current COVID VOC, namely, “Omicron,” “XBB,” “BA,” and “consensus,” “guideline,” “recommendation,” and “opinion” as major keywords, together with their MeSH and Emtree terms. In addition, we conducted a manual search through the review article references to identify any reported cases which might have been missed. The search and screening process were performed separately by two researchers, and the details of each step in the record retrieval and screening is presented in our PRISMA flow diagram,^12^ as depicted in Figure 1.

### 2.3 Eligibility criteria

Our inclusion criteria were original human studies or reports or recommendations or consensus opinions on prevention, vaccination, social measures, treatment options, and medication preferences regarding the current peak of COVID‐19. Exclusion criteria were in vitro studies, animal studies, basic science studies, and studies not including guidelines or recommendations for said concerns.

### 2.4 Screening and data extraction

After removal of duplicate records from the primary search results, two reviewers independently performed the Title‐Abstract screening on the retrieved records following the above inclusion and exclusion criteria. They then separately studied the full‐texts for the Title‐Abstract included articles carefully, to ascertain the eligibility and then started the data extraction. In cases of disagreement, they discussed the problem together, and if they could not resolve it, a third researcher with expertise in the field helped to reach a consensus opinion.

Our data extraction sheet contained the following columns: article reference, first author name, recommendation, and reasons.

## 3 ANSWERS TO HIGHLY CONCERNING QUESTIONS

### 3.1 How many COVID peaks have there been? How and when did the current peak start and how is it spreading in the world geographically? What are the incidence rates across countries? Which are the current peak's variants of interest (VOIs) and variants of concern (VOCs)?

The determination of the exact number of COVID‐19 peaks and waves detected to this date remains inconclusive, given the variations in the patterns and timings of COVID‐19 surges across different countries and regions.^13^ However, five major variants have been named since the inception of Sars‐CoV‐2 in 2019 up until 2021, including the Alpha, Beta, Delta, Gamma, and Omicron variants. Given the high transmissibility of the Omicron variant, it soon became the most dominant strain in late 2021 and early 2022 driving the fourth peak of COVID‐19 in United States and also globally. Since February 2022, the Omicron viruses have made up over 98% of the publicly available sequences, and they make the genetic source from which the newer SARS‐CoV‐2 variants have since emerged.^14^ Various subvariants of Omicron, including but not limited to BA.1, BA.5, BQ.1, and XBB.1.5, have been identified and disseminated, contributing to the mainstream subtypes of COVID‐19 spreaders.^15^

Omicron and its subvariants, solely, are known as the current peak's *VOCs* and the primary culprit for the latest uptick in cases.^8^ Most recently, as of January 2023 and April 2023, the XBB.1.5 and XBB.1.16 subvariants were announced as *variants of interest (VOIs)* by WHO, respectively.^8^ XBB.1.5 is considered to be the most transmissible strain of the virus so far detected in 54 countries, and multiple sequences of it have been reported in the United States, constituting 72.2% of all reportings. The remaining reports were in the United Kingdom at 7.3%, Canada at 5.0%, Germany at 2.7%, and Austria at 1.8%.^16^ One report from UK health Security Agency held XBB.1.5 responsible for 44% of all COVID‐19 cases.^17^

As of April 17, 2023, a total of 3648 sequences of the Omicron XBB.1.16 variant, which is also known as the “Arcturus,” the name of the brightest known star in the northern sky,^7^ have been documented across 33 countries. The majority of these cases (63.4%, 2314 sequences) were reported in India. The other countries include the United States (10.9%), Singapore (6.9%), and Australia (3.9%). Collectively, the existing data does not indicate that the Omicron descendent lineage XBB.1.16 poses a greater public health threat compared to XBB.1.5 and other currently circulating lineages.^18^

### 3.2 What are the differences between the current peak and the previous ones in terms of the factors below?

**Figure 2:**
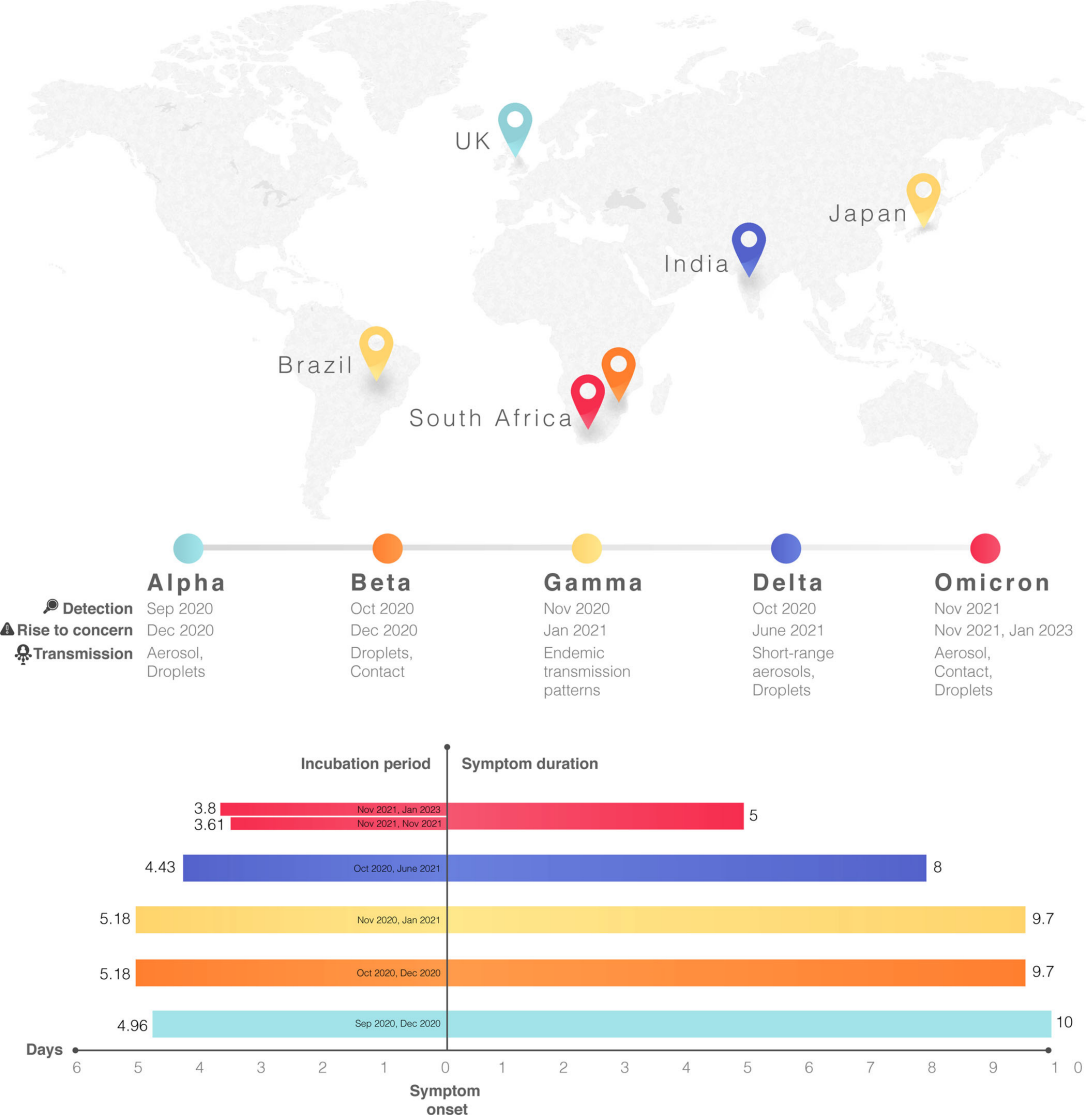
A comparison of major COVID waves. Illustrated here in three panels is the comparison of Alpha, Beta, Gamma, Delta, and Omicron waves regarding the place first detected on top panel, timeline of detection, rise to concern, and transmission pathways in middle table, and on the bottom bar chart the incubation period, symptom onset and symptom duration. The timeline of detection and rise to concern for each wave is also written on the respective bars. This figure is meant to accompany Table 1 data.

To answer this question, we have gathered data on the five major COVID waves, presented in Table 1, and depicted in Figure 2, on the most important aspects of the pandemic, namely, time of detection, country found, time of rise to concern, most common signs and symptoms, pathway of transmission, median incubation period, median duration of symptoms, long‐lasting symptoms (long COVID), cumulative percentage of population fully vaccinated, hospitalization risk, ICU admit percentage, and total deaths per 1 million.

**Table 1:** Comparison of the major COVID variants.

Variants	Alpha	Beta	Gamma	Delta	Omicron	
Time of detection	September 2020	October 2020	November 2020	October 2020	November 2021	
Country found	UK^19^	South Africa^20^	Brazil^21^ or Japan^22^	India^23^	South Africa^24^	
Time of rise to concern	December 2020	December 2020	January 2021	June 2021^23^	November 2021	January 2023
Most common signs and symptoms	Tiredness, headache, muscle ache, sneezing, congestion, sore throat, runny nose, loss or change of sense of smell or taste, persistent cough,^25^ and brain fog^26^	Similar to other variants, lighter symptoms which were outweighed by the Delta variant^27^	Headache, sore throat, runny nose, and fever^28^, ^29^	Asymptomatic, headache, sore throat, runny nose, fever,^29^ and loss or change of sense of smell or taste^25^	Asymptomatic, body ache, cough,^30^ fainting,^31^ fatigue,^32^ fever,^25^ headache, and runny nose^33^	A high fever (higher than previous variants), cough, conjunctivitis with pruritus, scratchy throat, runny nose, fatigue, body aches, headache, and congestion^34^
Pathway of transmission	Aerosol and droplets^35^	Droplets and contact^36^	Endemic transmission patterns^37^	Short‐range aerosols and droplets^38^	Aerosol, contact and droplets^39^	
Median incubation period	4.96 days^40^	5.18 days^40^		4.43 days^40^	3.61 days^40^	3.8 days^41^
Median duration of symptoms	Hospital LOS: 10 (7–13) days^42^	Pre‐Delta: 9.7 (9.0–10.4) days^43^		8·0 days (IQR 5.0–12.0)^44^	5.0 days (IQR 3.0–9.0)^44^	
Long‐lasting symptoms (long COVID)	6.3% (among adults in the United States)^45^ 35.9% (among health care workers in Italy)^46^	16.1% (among adults in Bangladesh)^47^	NM	7.4% (among triple vaccinated adults in the United Kingdom)^48^ 17.5% (among adults in the United States)^45^	9.1% for Omicron BA.2 (among triple vaccinated adults in the United Kingdom)^48^ 10.9% (among adults in the United States)^45^	11% (among adults in the United States)^49^
Cumulative percentage of world population fully vaccinated^50^	0.00% (up to December 31, 2020)		5.58% (up to May 31, 2021)	38.13% (up to October 31, 2021)	63.25% (up to December 31, 2022)	64.42% (up to June 16, 2023)
Hospitalization risk worldwide (hazard ratio)^51^	1.64 (1.29–2.07)	2.85 (1.56–5.23)	3.20 (2.40–4.26)	2.28 (1.56–3.34)	0.92 (0.56–1.52)	
ICU admit percentage and adjusted Hazard Ratio (in Peru^52^)	38.1% 1.43 (0.73‐2.81) (Alpha and Zeta)	NM	44.1% 1.95 (1.40–2.71)	29.2 0.90 (0.59–1.37)	13.6% 0.46 (0.10–2.02)	NM
Total deaths per 1 Million in each time period^50^	244.10 (up to December 31, 2020)		288.28 (January 1, 2021 to May 31, 2021)	162.80 (June 1, 2021 to October 31, 2021)	205.29 (November 1, 2021 to December 31, 2022)	27.81 (January 1, 2023 to June 14, 2023)

Abbreviations: ICU, intensive care unit; IQR, interquartile range; LOS, length of stay; NM, not mentioned.

### 3.3 How effective has the previous vaccination been in preventing severe COVID, long COVID, and hospitalization, along with lowering death rates in the current peak?

To overcome this pandemic, vaccination has been an effective way to reduce disease spread. Over 50 COVID‐19 vaccine candidates have reached clinical trials, including AstraZeneca, Moderna, Pfizer, Sputnik, Johnson and Johnson, Sinopharm, Sinovac, Bharat, and so on.^53^ As of June 4, 2023, a total of 13.41 billion vaccine doses have been administered globally.^50^

After the initial vaccinations, the first case of mutant variant in the United States was reported on December 29, 2020 in Denver, Colorado and since then, there have been many reports of several other mutant variants globally.^54^ The emergence of these new variants imposes an important question onto the effectiveness of current vaccination on different variants of the COVID‐19 infection.

#### 3.3.1 Effectiveness of vaccines and boosters for preventing severe infection, symptomatic disease, and death

We will discuss prominent research on this topic. According to a comprehensive review by Rikin Patel et al., the BNT162b2 vaccine does not show any reduction in efficacy against variants with the N501Y mutation (which is present in Omicron, and previous B.1.351 [Beta] and B.1.1.7 [Alpha] strains^55^) relative to the original strain.^54^

According to a test‐negative design study, the BNT162b2 and mRNA‐1273 vaccines' effectiveness increased against Omicron SARS‐COV‐2 infections with the B.1.1.529 sublineage, with each additional dose, and for a fourth dose, the vaccines efficacy was found to be 49% against infection, 69% opposing symptomatic infection, and 86% countering severe patient outcomes.^56^ Other studies support the better performance of booster doses against severe outcomes as well.^57^

According to another study, vaccine effectiveness after the second, third, and fourth doses of BNT162b2 against omicron BA.1 and BA.2, were 13%, 48% and 69%, respectively, measured 7 days following immunization, which were then diminished to 7%, 26%, and 35%, respectively, at a 100 days after vaccine administration.^58^ Regarding CoronaVac's effectivity after the second, third, and fourth doses against Omicron BA.1 and BA.2, the vaccine was effective in 5%, 30%, and 56% of participants, respectively, 7 days following immunization, which was then reduced to 1%, 6%, and 11%, respectively, a 100 days after administration.^58^

According to a systematic review, several studies have demonstrated that a booster‐dose vaccine is effective countering SARS‐CoV‐2 variants, including Omicron.^59^ A study from the United States examined the association between three doses of BNT162b2 or the mRNA‐1273 vaccine and symptomatic SARS‐CoV‐2 infection resulting from Omicron and Delta variants and has found that when comparing triple vaccinated participants with an mRNA COVID‐19 vaccine, against unvaccinated participants or those having received two doses, triple vaccinated individuals were less likely to be symptomatic cases if infected with SARS‐CoV‐2 and were more protected from Delta rather than the Omicron variant.^60^

#### 3.3.2 Effect of vaccination on long COVID

According to a systematic review and meta‐analysis, two and above doses of vaccination given to those never priorly infected with SARS‐CoV‐2 were found to significantly decrease the rates of the appearance of long COVID.^61^, ^62^

The protective effect of vaccination differs against different symptoms of long COVID; that is, the risk of postacute complications in the metabolic and cardiovascular systems, in addition to the risk of mental disorders, was significantly decreased in the priorly vaccinated population.^63^

### 3.4 Who are the high‐risk groups for transmission, being asymptomatic carriers, having severe disease or mortality in the current peak?

#### 3.4.1 Mortality, morbidity, and severity

According to Zhang et al., older age, male sex, underlying comorbidities, disparities in race or ethnicity, and working in healthcare are the known risk factors for adults' susceptibility to COVID‐19.^64^ Patients above 70 years of age show a 65% higher risk for COVID‐19.^65^

Male sex, as discussed above, is an important risk factor with evidence demonstrating that men had an increased risk of infection, severity of disease, admission to the ICU, and mortality when compared to women. Altogether, men had an 8% higher risk of infection with COVID‐19 when compared to women.^64^, ^65^, ^66^ Underlying comorbidities also pose a risk for severity, mortality, and morbidity with COVID‐19. Evidence has showed that cardiovascular diseases were the most common underlying comorbidities in all phases of the COVID‐19 pandemic.^67^ Among those with malignancies, patients with hematological cancers are at a higher risk of COVID‐19 compared to those with solid malignancies, which might be explained by the elevated expression of cellular receptors.^68^

#### 3.4.2 Transmission and asymptomatic carriers

Regarding asymptomatic carriers, according to a study, no significant difference was found between the sexes in asymptomatic carriers. In age subgroups, asymptomatic carriers were mainly children (49.6%), followed by adults (30.3%) and the elderly (16.9%).^69^ In another study, a higher percentage of asymptomatic infections was observed in pregnant women, nursing home residents or staff, and travelers by air or cruise.^70^ As for the high‐risk groups in terms of transmission, children's transmission of SARS‐COV‐2 seems to grow higher as new variants emerge,^71^ with this being in unison with the previously mentioned studies.

### 3.5 What are the recommendations and consensus opinions on the current peak?

Regarding the COVID‐19 pandemic in 2023, we have conducted a systematic review on the latest recommendations and consensus opinions for preventive measures, new treatment and prophylactic options, booster vaccinations, and specific notes for at‐risk groups. As we move forward with COVID‐19, especially with the new PHE declaration,^10^, ^72^ it is important to implement the latest recommendations and guidelines for the current peak. We have gathered an organized set of recommendations for all different aspects of COVID‐19, together with the reasons behind the recommendations. The methods are discussed in the methods section and the resulting data are categorized by the recommendations on booster vaccination, preventive, and treatment measures, in both the general population and at‐risk groups, presented in Table 2, in these four sections.

**Table 2:** Recommendations and consensus opinions.

Reference	First author	Recommendations	Reasons
1. Preventive measures in the general population			
[73]	Arashiro, T.	1. Not relaxing social and public health measures for vaccinated people (certificates, passports, etc.) 2. Continuous communication of the government with the public regarding the continuation of infection prevention measures (wearing masks, physical distancing, etc.) even after vaccination	Preventing vaccinated people from engaging in high‐risk behaviors, discontinuing protective measures, and spreading the disease
[74]	Kim, Y.Y.	Any number or type of COVID‐19 vaccinations, especially in people at risk of serious illness	Vaccination remaining the most important modifiable intervention to protect public health and preventing death
[75]	Kompaniyets, L.	Using Ad26. COV2.S+mRNA vaccines (two doses total) or three mRNA vaccine doses instead of two Ad26.COV2.S doses	1. Better overall results 2. Reducing less (COVID‐19 diagnosis, outpatient encounter, etc.) or more severe (ICU admission, MV, etc.) outcomes
[76]	Lin, Y.F.	1. Dynamically adjusting social restrictions (mass gathering limitations, school closures, social distancing, etc.) via pilots and implementing feasible ones instead of relaxing the measures entirely 2. Encouraging people to change their personal hygiene habits (wearing masks, washing hands, etc.) and take antigen self‐tests frequently as alternatives to restrictive policies 3. Balancing restrictions with their impacts on the daily life and economy by reducing contact to a rational amount through graded or subregional limitations 4. Improving the construction of quarantine chambers and home quarantine guidelines	1. Preventing the disease from spreading and overwhelming the health system (hospital bed shortage, etc.) 2. Reducing the number of COVID‐19 infections and preventing later adverse outcomes 3. More feasibility in longer period of time 4. Minimizing secondary transmission
[77]	Wu, Y.	1. Withdrawing isolating limitations for test‐positive symptomatic patients and shortening the quarantine period for their close contacts 2. Repeat testing not recommended for indication of patients being infectious and adjusting the prevention and control strategies based on the duration of viable virus shedding	1. Shortened duration of viable virus shedding in Omicron variant 2. Significantly longer period of virus RNA shedding compared to the viable virus shedding (false positives)
2. Treatment and prophylaxis in the general population			
[78]	Aleem, A.	1. Using antiviral (molnupiravir, nirmatrelvir+ritonavir, and remdesivir) and antibody‐based (bamlanivimab+etesevimab, casirivimab+imdevimab, sotrovimab, and bebtelovimab) medications during the early phase of disease 2. Using anti‐inflammatory (dexamethasone) and/or immunomodulatory (baricitinib, tocilizumab, ruxolitinib, and tofacitinib) medications during the late phase of disease 3. Not using medications without efficacy, including HCQ, CQ, lopinavir+ritonavir, ivermectin, monoclonal antibodies, interferon β‐1a, IL‐1 inhibitors, sarilumab, and siltuximab 4. Using tixagevimab+cilgavimab for pre‐exposure prophylaxis in adults and pediatrics (≥12 y/o with ≥40 kg weight) without current evidence of COVID‐19, no recent exposure to COVID‐19‐positive individuals, and: (a) Moderate or severe immunocompromised status due to a medical condition or medication, (b) using immunosuppressive medications causing a probability of inadequate COVID‐19 vaccine response, or (c) contraindication of COVID‐19 vaccination due to a past adverse reaction	1. Halting the fast replication of the virus before or soon after the onset of symptoms 2. Inhibiting hyperinflammatory and prothrombic state resulting in improvement of signs, symptoms, and mortality rate 3. No available supporting evidence or no significant improvement in any of outcomes, especially for newer SARS‐CoV‐2 variants 4. Neutralizing SARS‐CoV‐2 virus by binding to its specific spike proteins
[79]	Beaumont, A.L.	1. In outpatients, nosocomial cases, and patients hospitalized for other conditions (no oxygen requirement): (a) Symptomatic management (analgesics, antipyretics, etc.), isolation, educating on when to contact a physician, evaluating severe disease risk, no treatment in the absence of risk factors; (b) Nirmatrelvir+ritonavir (100‐300 mg BID for 5 d, within first 5 d of symptoms onset) if any risk factor present; (c) Remdesivir (200, 100, 100 mg IV QD for 3 d if eGFR ≥ 30 mL/min) as second line; (d) monoclonal antibodies not recommended; (e) Convalescent plasma discussed case‐by‐case 2. In inpatients (with oxygen requirement): (a) Conventional oxygen therapy, dexamethasone (or equivalents, 6 mg QD for 10 d or until discharge, whichever sooner), remdesivir (for 5 d or until discharge, within 10 d of symptoms onset), and tocilizumab or baricitinib (if oxygen requirement rapidly increasing and systemic inflammation present); (b) high‐flow oxygen or MV, dexamethasone, and immunomodulators (tocilizumab, baricitinib, etc.)	1. Effectivity of the treatments based on supporting evidence 2. Preventing hyperinflammatory state and acute respiratory failure
[80]	Elsaghir, H.	1. No monoclonal antibody treatment indicated for the treatment of COVID‐19 2. Using tixagevimab+cilgavimab for pre‐exposure prophylaxis 3. In outpatients with mild to moderate COVID‐19 (no oxygen supplement): nirmatrelvir+ritonavir (within first 5 d of symptoms onset), remdesivir (for 3 d), and molnupiravir (if both unavailable or contraindicated)	1. Limited efficacy against the current dominant variants 2. The only monoclonal antibody currently authorized for COVID‐19 3. Current recommendations in the absence of monoclonal antibodies
[81]	Imran, L.	Using nirmatrelvir+ritonavir in patients with mild to moderate COVID‐19 and high risk of progression to severe form within the first 5 d of symptoms onset	Decreased hospitalization and mortality
[76]	Lin, Y.F.	1. Monitoring arterial oxygenation using pulse oximetry in the patients diagnosed with COVID‐19 at home and presenting for care when hypoxemia is detected 2. Customizing treatments based on different symptom severities	1. Decreasing the risk of complications in patients with severe hypoxemia in the absence of dyspnea 2. More effective and feasible treatments
[82]	Saravolatz, L.D.	Using nirmatrelvir+ritonavir (in ≥12 y/o patients with ≥40 kg weight) or molnupiravir (in ≥18 y/o patients and not recommended in transplant recipients and pregnant women) for the 5 d long outpatient treatment of mild to moderate COVID‐19 within the first 5 d of symptoms onset	1. Decreasing hospitalization and mortality (nirmatrelvir+ritonavir being more efficient) with rare, mild adverse effects (except possibility of mutation in pregnancy or bone and cartilage toxicity if aged <18 y/o caused by molnupiravir)
[83]	Sun, L.Q.	1. Early use of antivirals in infected patients 2. Using nirmatrelvir+ritonavir, remdesivir, and molnupiravir in mild cases 3. Removing monoclonal antibodies and convalescent plasma from guidelines for newer SARS‐CoV‐2 variants 4. Oxygen therapy (HFNC, NIV, MV, ECMO, etc.) and prone position ventilation if hypoxemia is present 5. Using anticoagulants in infected patients 6. Using immunomodulators (glucocorticoids, IL‐6 receptor inhibitors, and JAK inhibitors) in severe or critical cases 7. Using glucocorticoids and baricitinib in moderate cases	1. Sooner inhibition of viral replication and the subsequent damage to cells and tissues 2. Effectiveness of these antiviral medications 3. Decreased effectiveness of these medications in the newer variants 4. Improving disease outcomes 5. Reduction in the need for organ support and progression to intubation and death 6 and 7. Preventing secondary immune damage by suppressing excessive inflammatory response
3. Booster vaccination for the general population			
[78]	Aleem, A.	Using third vaccine dose (booster injection)	Higher protection levels and halting the decrease in immunity
[73]	Arashiro, T.	Using an mRNA booster vaccine after the primary series	Significant increase in VE
[84]	Brosh‐Nissimov, T.	1. Using a fourth mRNA vaccine (second booster) ≥4 m after the third injection (first booster), particularly in ≥60 y/o individuals, immunocompromised patients, and healthcare personnel 2. Third mRNA vaccine (first booster) injection in younger, healthier population	1. Significant decrease in infection, deterioration to severe disease, MV requirement, and death 2. Preventing hospitalization and adverse outcomes
[85]	Gaviria‐Agudelo, C.	1. Injecting a bivalent booster vaccine ≥2 m after the previous vaccination, regardless of it being the second vaccine from the primary series or being a monovalent booster vaccine in individuals aged ≥12 y/o (using Pfizer vaccine) and ≥18 y/o (using Moderna vaccine) 2. Delaying vaccination for 3 m after a recent COVID‐19 infection 3. Separating COVID‐19 and influenza vaccines by ≥1 inch if administered at the same visit and injecting them in different limbs if a higher dose or adjuvanted vaccine is being administered (e.g., in elderly individuals)	1. Increased immunity against older and newer SARS‐CoV‐2 variants 2 and 3. Decreasing the risk of potential adverse effects
[86]	Hause, A.M.	1. Injecting a bivalent booster dose ≥2 m after the primary series or monovalent booster vaccination in 5–11 y/o (using Pfizer‐BioNTech vaccine) and 6–17 y/o (using Moderna) children 2. Injection of age‐appropriate bivalent mRNA booster dose in all individuals aged ≥6 m	Efficacy of bivalent COVID‐19 vaccines and rare adverse effects in children
[87]	Link‐Gelles, R.	Using bivalent mRNA booster vaccines when eligible in persons who have received 2–4 monovalent vaccines	Extra protection of bivalent vaccines against symptomatic XBB/XBB.1.5 variants' infection for ≥3 m after vaccination
[88]	Lu, P.J.	1. Injection of bivalent booster vaccine in ≥12 y/o patients 2. Using a network of trained messengers trusted by local communities to promote booster vaccination and their safety while reducing barriers to vaccination	1. Maintaining protection against COVID‐19 and reducing risk for severe adverse outcomes 2. Spreading booster vaccination using culturally appropriate messaging and decreasing misinformation
[89]	Petrie, J.G.	1. Injection of booster vaccines, particularly bivalent types 2. Not recommending injection of second monovalent mRNA booster vaccine in adults aged ≥50 y/o	1. Waning immunity after the primary series and poor response of monovalent vaccines to newer SARS‐CoV‐2 variants 2. No significant increase in immunity against newer variants
[90]	Sinclair, A.H.	1. Injecting bivalent booster vaccine in all eligible adults 2. Increasing bivalent booster coverage using evidence‐based strategies (trusted messengers, etc.) to convey information about booster vaccines and waning immunity of previous vaccinations while improving vaccine availability	1. Waning immunity of the previous vaccinations and their poor response against newer SARS‐CoV‐2 variants 2. Need for awareness about eligibility to receive booster vaccines and their efficacy and safety
[91]	Solante, R.	1. Using three‐dose primary series of vaccines and more regular booster vaccine injections with shorter intervals (4–6 m) based on risk of COVID‐19 infection (healthy, elderly, immunocompromised, etc.) 2. Focusing on improving coverage of available COVID‐19 vaccines instead of developing newer ones	1. High efficacy and waning immunity of current COVID‐19 vaccines even against newer variants 2. High rate of mutation and distraction from the immediate needs
[92]	Stultz, J.S.	Vaccination of children aged ≥6 m with age‐appropriate mRNA or protein‐based vaccines as the primary series, followed by a bivalent mRNA booster	Reported efficacy and safety in the literature
[93]	Wang, M.L.	Using mRNA booster vaccines (preferably ≥2 boosters) in individuals aged ≥12 y/o who have received their primary series ≥5 m before	Immunity against newer variants of SARS‐CoV‐2
4. Prevention, booster vaccination, prophylaxis, and treatment for at‐risk groups			
4.1. Those with immune conditions or reactions to vaccines			
[94]	Convertino, I.	Considering tixagevimab+cilgavimab cocktail as an alternative medication for COVID‐19 prophylaxis or passive immunization in patients that cannot be vaccinated (due to adverse effects or immune conditions)	Good protective results, particularly in pre‐Omicron variants of SARS‐CoV‐2
[85]	Gaviria‐Agudelo, C.	Injecting EvuSHELD monoclonal antibodies every 6 m in ≥12 y/o individuals with moderate to severe immunocompromising conditions or a history of severe adverse reaction to COVID‐19 vaccines or their components	Passive immunity against COVID‐19
[95]	Rescigno, M.	Injection of fourth and fifth COVID‐19 vaccines (boosters) in immunocompromised patients	Improved immunity against COVID‐19 due to increase in T‐cell response, even in the absence of humoral response in patients receiving B‐cell targeted therapies
4.2. Transplant recipients			
[96]	Boutin, C.A.	1. Injecting a bivalent booster ≥2 m after the initial vaccines in all transplant patients 2. Completing all vaccinations ≥2 w before transplantation 3. Discontinuing usage of tixagevimab+cilgavimab and focusing on other preventive strategies 4. Face masking, hand hygiene, social distancing, and avoiding crowded indoor activities in transplant patients 5. PCR‐based testing in asymptomatic transplant donors or diagnosing symptomatic transplant patients 6. Any COVID‐19‐positive specimen at the time of death being a contraindication for lung donation 7. Small bowel donation by COVID‐19‐positive individuals not recommended 8. No restriction in transplanting organs other than lungs and intestines from COVID‐19‐positive donors 9. No recommendation of antiviral medications or monoclonal antibodies in the recipients 10. Transplanting organs from donors with COVID‐19‐associated or vaccine‐associated clots not recommended 11. Delaying all elective surgeries, including living donation, in positive patients for 7 w until symptoms resolved	1 and 2. Increasing immune response considering the immunocompromised state 3. Monoclonal antibodies being ineffective against newer SARS‐CoV‐2 variants 4. Effective nonpharmacological approaches against COVID‐19 5. Screening and early treatment of COVID‐19 6. Significant risk of transmission of COVID‐19 to the recipient and/or healthcare workers 7. Presence of SARS‐CoV‐2 in the intestinal lymphoid tissue and stool (especially in children) 8. No evidence of transmission 9. No supporting evidence available 10. Transmission of hypercoagulability from COVID‐19 donors 11. Decreasing postoperative mortality
[97]	Park, J.K.	1. Routinely checking vaccine response in KTRs after 3–4 doses and using monoclonal antibodies or alternative protective measures if needed 2. Different alternatives if inadequate response perceived: (a) additional booster doses (up to six doses total); (b) primary prophylaxis using monoclonal antibodies; (c) lowering the dosage of mycophenolate mofetil and/or replacing it with belatacept 3. Early administration of monoclonal antibodies in KTRs with mild COVID‐19	1. Persistently weak or absent response to COVID‐19 vaccines in immunocompromised patients despite booster injections 2. Maintaining sufficient immunity in KTRs 3. Causing passive immunity
[82]	Saravolatz, L.D.	Using sotrovimab or remdesivir in transplant recipient COVID‐19 outpatients	No evidence regarding the safety of nirmatrelvir+ritonavir or molnupiravir in immunocompromised patients
[98]	Zhang, L.	Using a third homologous inactivated mRNA vaccine (booster dose) in KTRs	Significant improvement of cellular (but not humoral) immunity against ancestral variants of COVID‐19, but poor protection against Delta and Omicron variants
4.3. Patients with cancer			
[99]	Giesen, N.	1. COVID‐19 vaccination before starting cancer therapy, if possible, but not pausing ongoing cancer therapy for vaccination 2. Simultaneous vaccination against influenza in patients with cancer if the COVID‐19 vaccination schedule overlaps with the seasonal flu vaccination schedule 3. Moderate recommendation of using tixagevimab+cilgavimab as pre‐exposure prophylaxis in cancer patients with high risk of poor vaccine response or COVID‐19 exposure 4. Strong recommendation of using casirivimab+imdevimab as postexposure prophylaxis in unvaccinated or poorly responding patients with cancer 5. Strong recommendation of monoclonal antispike antibodies for early treatment of outpatients with cancer, particularly unvaccinated or poorly responding ones 6. Using convalescent plasma as prophylaxis not recommended, but marginally recommended after COVID‐19 symptoms onset 7. Strong recommendation of using nirmatrelvir+ritonavir in outpatients with cancer while paying attention to possible adverse drug interactions with ritonavir 8. Moderate recommendation of remdesivir in outpatients with cancer 9. Marginal recommendation of molnupiravir in outpatients with cancer 10. Moderate recommendation of remdesivir (for ≤10 d) in hospitalized patients with cancer and COVID‐19 and not‐requiring MV (not in patients with MV) 11. Using remdesivir, IL‐6 inhibitors, or JAK inhibitors within the first 24 h of ICU admission even if using MV 12. Using dexamethasone, IL‐6 monoclonal antibodies, JAK inhibitors, and other immunosuppressors in hospitalized patients with cancer and COVID‐19 (oxygen support not contraindicated) 13. Strong recommendation of dexamethasone (6 mg/d for 10 d) if oxygen support needed 14. Adding anti‐IL‐6 monoclonal antibodies (tocilizumab, sarilumab, etc.) or JAK inhibitors (baricitinib, tofacitinib, etc.) to dexamethasone in patients with systemic inflammation (e.g., elevated CRP) and oxygen support (not MV) 15. Strong recommendation of thromboembolic prophylaxis with low‐dose LMWH in hospitalized patients with cancer and moderate to severe COVID‐19 16. Using primary and booster vaccines, particularly mRNA and/or bivalent vaccines with highest possible dosage without restriction based on respective local guidelines and availability in patients with cancer 17. Prioritizing patients with cancer for booster vaccination	1. Decreased and shorter immune response to COVID‐19 vaccines in patients with cancer and no evidence of vaccines inducing cancer relapse 2. No interaction between COVID‐19 and influenza vaccines and susceptibility of cancer patients to flu 3–5. Efficacy of monoclonal antibodies against COVID‐19 in the absence of vaccine‐induced immunity, particularly against pre‐Omicron COVID‐19 variants 6. No prevention of COVID‐19 and mixed results in treatment 7. Efficacy against COVID‐19 even in its newer variants 8. Lower potential of adverse drug interactions compared with ritonavir 9. Lower efficacy and limited supporting evidence 10. Efficacy against COVID‐19 if MV not required 11. Prevalent rapid deterioration in cancer patients 12. Increased mortality based on large studies 13 and 14. Significant improve in outcomes and mortality 15. Increased risk of thromboembolism 16 and 17. (a) High prevalence of elderly patients with cancer and comorbidities that decrease VE; (b) sooner decrease in immunity after each dose compared to general population; (c) high prevalence of immunosuppressive therapies that decrease response to vaccines
[100]	Thakkar, A.	1. Using third and preferably fourth vaccines (boosters) in patients with cancer 2. Testing the serological and cellular markers of response to vaccines in cancer patients	1. Improved immunity against COVID‐19 due to increase in T‐cell response, even in the absence of humoral response in patients receiving B‐cell targeted therapies; b. Significantly stronger response after the fourth vaccine in low seropositive patients and even seroconversion in some of nonresponders 2. Identifying the at‐risk patients for further prophylactic and preventive measures
4.4. Elderly			
[76]	Lin, Y.F.	Extending the duration of immunity against COVID‐19 using additional vaccine doses and widely available oral antiviral medications in hospitals, pharmacies, community healthcare centers, and so on, particularly for >60 y/o individuals	The necessity of special attention to the elderly population
[93]	Wang, M.L.	Using a fourth mRNA vaccine dose (booster) in elderly individuals with immunosuppression or in need of long‐term care	Immunity against newer variants of SARS‐CoV‐2
4.5. Pregnancy			
[101]	Corsi Decenti, E.	Recommendation of primary vaccination and booster doses at any time in gestation	Higher risk of moderate to severe COVID‐19, urgent or emergent cesarean section, and preterm birth in unvaccinated pregnant women compared to the vaccinated group
[102]	Lipschuetz, M.	Injecting a third mRNA vaccine (booster) during pregnancy	Decrease in incidence rate and duration of early (≤4 m of life) infant hospitalization due to COVID‐19 compared to receiving only a second dose of vaccine ≥5 m before delivery
4.6. Candidates for surgery			
[103]	Barie, P.S.	1. Patients are recommended to be free of active infection at the time of surgery 2. Pre‐Omicron guidelines for elective surgery delay: (a) 4 w if asymptomatic or recovering from mild, nonrespiratory symptoms; (b) 6 w if recovering from cough or dyspnea without hospitalization; (c) 8–10 w if hospitalized or symptomatic with diabetes or immunocompromised state; (d) 12 w if recovering from critical COVID‐19 3. Using intensified thromboprophylaxis in patients who must undergo elective surgery in the early postrecovery period (from perioperation until 1 m postoperation)	Decreasing risk of adverse outcomes, including respiratory failure, pulmonary embolism, sepsis, and mortality

*Note*: Any unspecified vaccine in this table is COVID‐19 vaccine (any kind), and any unspecified diagnosis is infection with SARS‐CoV‐2 (any variant).

Abbreviations: BID, twice a day; CQ, chloroquine; CRP, C‐reactive protein; d, day[s]; ECMO, extracorporeal membrane oxygenation; eGFR, estimated glomerular filtration rate; h, hour[s]; HCQ, hydroxychloroquine; HFNC, high‐flow nasal cannula; ICU, intensive care unit; IL, interleukin; IV, intravenous; JAK, Janus kinase; kg, kilogram[s]; KTR, kidney transplant recipient; LMWH, low molecular weight heparin; m, month[s]; mL/min; milliliter[s] per minute; [m]RNA, [messenger] ribonucleic acid; MV, mechanical ventilation; NIV, noninvasive ventilation; PCR, polymerase chain reaction; mg, milligram[s]; QD, once daily; VE, vaccine efficacy; w, week[s]; y/o, year[s] old.

(1) Preventive measures in the general population: what to do to prevent transmission, together with social or personal guidelines to lower the risk of infection are presented.
(2) Treatment and prophylaxis in the general population: both inpatient and outpatient treatment options: which previous treatment options are not recommended anymore and which new options are preferred now are presented.
(3) Booster vaccination for the general population: how many booster doses are recommended and which vaccines are preferred, for both children and adults are presented.
(4) Prevention, booster vaccination, prophylaxis, and treatment for at‐risk groups: specific recommendations and consensus on interventions for those at risk, presented in six categories: those with immune conditions or reactions to vaccines, transplant recipients, patients with cancer, elderly, pregnancy, and candidates for surgery.

## 4 DISCUSSION

With the current rise of the new Omicron subvariants, we have gathered answers to the most important questions regarding the COVID pandemic. Regarding effectiveness of vaccination, SARS‐COV‐2 vaccines could effectively reduce the mortality, severe cases, and symptomatic cases resulting from SARS‐COV‐2, and provide a modest protection against infection.^54^, ^56^

To discuss vaccine effectiveness against different COVID genetic variants, no reduction was seen in the efficacy of the BNT162b2 vaccine against mutant strain N501Y (present in Omicron, and previous B.1.351 [Beta] and B.1.1.7 [Alpha] variants^55^), when compared to the original strain.^54^ However, concerns for the high potential of the new XBB and BQ.1 sublineages for vaccination breakthrough still remain.^104^ A study showed that bivalent vaccination can mostly protect against the BQ.1.1 strain, but does not provide satisfactory protection against the XBB.1 and XBB.1.5 strains in those with cancer,^105^ putting more emphasis on the protective measures and recommendations discussed in Table 2 regarding patients at risk.

With regard to the efficacy of the two most commonly known vaccines, Pfizer and Moderna, on previously concerning strains, studies showed that the Pfizer vaccine was effective in all ages against asymptomatic and symptomatic infection, as well as hospitalization and mortality, including infection with the B.1.1.7 (Alpha) variant.^106^ On the other hand, several research studies in different countries have shown a decline in efficacy against the Delta variant, B.1.617.2.^107^ In the United Kingdom, there was 35.6% efficacy demonstrated after the first dose of vaccination and 88% efficacy after the second dose against the B.1.617.2 (Delta) variant for preventing symptomatic disease.^108^ The Pfizer vaccine was less effective in stopping infection, but was able to prevent the worst COVID outcomes of hospitalization and mortality.^109^

Regarding the Moderna mRNA‐1273 vaccine, it is found to be 94.1% effective at protecting against symptomatic COVID‐19 after a double‐dose vaccination.^110^ Serum neutralizing activity of the Moderna vaccine was measured against the B.1.1.7 (Alpha) and B.1.351 (Beta) variants.^111^ Results indicate that the vaccine maintains its neutralizing activity against the B.1.1.7 (Alpha) variant. However, a decrease in titers of neutralizing antibodies was reported against the B.1.351 (Beta) variant. Protection against the B.1.351 (Beta) variant by the Moderna mRNA vaccine remains to be seen.^110^, ^111^ Moderna vaccine efficacy against the Delta variant, B.1.617.2 is 76%. Despite its lower efficacy against acquiring the infection, the vaccine has maintained a 90‐95% efficacy against hospitalization and death.^112^

With regard to long COVID, different studies report varying percentages for each peak,^113^ which could be due to different definitions of long COVID, or different study settings or time from participants' vaccinations.^114^ However, it does appear that long COVID rates are declining as we move forward, mainly due to higher percentages of vaccinated and fully vaccinated individuals.^115^, ^116^

Regarding high‐risk groups for transmission and asymptomatic carriers, elderly individuals usually have more comorbidities and weaker immunity against infections, therefore older age is a risk factor for mortality, morbidity, and severity of COVID‐19 infection. In addition, the different effects of hormones in inflammatory processes, different levels of cell receptors, and contrast in lifestyle in males may account for their predisposition to COVID‐19 infection. As SARS‐COV‐2 infection is capable of inducing endothelial inflammation, patients with cardiovascular disorders could be more susceptible to COVID‐19 infection.^117^ Among different subgroups, children have the most asymptomatic cases among them and their transmission of SARS‐COV‐2 has risen with the new variants.^71^

Regarding recommendations and consensus opinions, for which we have conducted a systematic review, for preventive measures, the consensus was on not relaxing social and public health measures for vaccinated people,^73^ while encouraging any number or type of COVID‐19 vaccinations, especially in people at risk of serious illness.^74^ For treatment and prophylaxis in the general population, the consensus option for antiviral treatment was combination therapy using Nirmatrelvir and Ritonavir,^78^, ^79^, ^80^, ^81^, ^82^, ^83^ and the consensus for pre‐exposure prophylaxis was Tixagevimab and Cilgavimab combination.^78^, ^80^ As for booster vaccinations, a third or fourth booster have been widely recommended,^78^, ^91^ particularly in individuals above 60, immunocompromised patients, and healthcare personnel.^84^ A bivalent mRNA booster vaccine has also been recommended when eligible^86^, ^88^, ^90^ in persons who have received 2–4 monovalent vaccines.^87^ And for the highly at‐risk groups, the recommendations in Table 2, can be of utmost importance when properly implemented, helping protect individuals more prone to severe disease, morbidity, and mortality.

## 5 CONCLUSION

The current state of the COVID‐19 pandemic and the Omicron variant in 2023, is characterized by new cases, deaths, and concerns about where the pandemic is heading. The worldwide situation is complex, with new VOC emerging and raising concerns about the efficacy of vaccines and currently known immunotherapies. It is crucial to continue monitoring the situation, implementing strict interventions, and adhering to strategies that the best evidence addresses the ongoing challenges posed by the pandemic.

## AUTHOR CONTRIBUTIONS

Contributions to the current study are Homa Pourriyahi, and Azadeh Goodarzi in the design; Homa Pourriyahi and Nima Hajizadeh in database search, screening publications, literature review, quality evaluation, and bias assessment; Homa Pourriyahi, Nima Hajizadeh, Mina Khosravi, Homayoun Pourriahi, Sanaz Soleimani, Nastaran Sadat Hosseini, and Arash Pour Mohammad in preparing the draft for the manuscript; Nastaran Sadat Hosseini in designing, drawing and curing an original figure (Figure 2); and Azadeh Goodarzi in drafting, checking, and revising the manuscript critically based on their expertise for important intellectual content. All authors have read and approved the final version to be published and agreed to be accountable for all aspects of the work. All authors agreed on the order in which their names are listed in the manuscript.

## CONFLICT OF INTEREST STATEMENT

The authors declare no conflict of interest.

## ETHICS STATEMENT

Patients or the public were not involved in the design, or conduct, or reporting, or dissemination plans of our research.

## Supporting information

Supporting information.

## Data Availability

All data supporting the findings of the present study are available upon reasonable request from the corresponding authors.
