# Supplementary material for: New variants of COVID‐19 (XBB.1.5 and XBB.1.16, the “Arcturus”): A review of highly questioned concerns, a brief comparison between different peaks in the COVID‐19 pandemic, with a focused systematic review on expert recommendations for prevention, vaccination, and treatment measures in the general population and at‐risk groups
Source: Immun Inflamm Dis. 2024 Jun 27;12(6):e1323. doi: 10.1002/iid3.1323 (PMC11211615; doi:10.1002/iid3.1323)
Supplement: Supplementary file 1 — Supporting information. [file IID3-12-e1323-s001.docx]

**New variants of COVID-19 (XBB.1.5 and XBB.1.16, the “Arcturus”): A systematic review of what the best evidence says about highly questioned concerns, with focus on expert recommendations and a brief comparison between different peaks in the COVID-19 pandemic**

| Database | Search string | Number of results |
| --- | --- | --- |
| Pubmed | ((Omicron) OR (XBB.1.5) OR (BQ.1.1) OR (BQ.1.) OR (BA.5) OR (CH1.1) OR (XBB) OR (B.1.617.2) OR (BA.4) OR (B.1.1.529) OR (BA.2) OR (BA.2.12.1) OR (BA.2.75) OR (BN.1) OR (XBB.1.9.1) OR (BA.4.6) OR (BA.5.2.6) OR (BF.7) OR (BF.11) OR (XBB.1.5.1) OR (XBB.1.5) OR (BA.2.75.2) OR (BN1)) AND ((Guideline) OR (Expert opinion) OR (Recommendation) OR (Consensus) OR (Opinion)) | 109 |
| Scopus | TITLE-ABS-KEY(((Omicron) OR (XBB.1.5) OR (BQ.1.1) OR (BQ.1.) OR (BA.5) OR (CH1.1) OR (XBB) OR (B.1.617.2) OR (BA.4) OR (B.1.1.529) OR (BA.2) OR (BA.2.12.1) OR (BA.2.75) OR (BN.1) OR (XBB.1.9.1) OR (BA.4.6) OR (BA.5.2.6) OR (BF.7) OR (BF.11) OR (XBB.1.5.1) OR (XBB.1.5) OR (BA.2.75.2) OR (BN1)) AND ((Guideline) OR (Expert opinion) OR (Recommendation) OR (Consensus) OR (Opinion))) | 49 |
| Web of Science | TS=((Omicron) OR (XBB.1.5) OR (BQ.1.1) OR (BQ.1.) OR (BA.5) OR (CH1.1) OR (XBB) OR (B.1.617.2) OR (BA.4) OR (B.1.1.529) OR (BA.2) OR (BA.2.12.1) OR (BA.2.75) OR (BN.1) OR (XBB.1.9.1) OR (BA.4.6) OR (BA.5.2.6) OR (BF.7) OR (BF.11) OR (XBB.1.5.1) OR (XBB.1.5) OR (BA.2.75.2) OR (BN1)) AND ((Guideline) OR (Expert opinion) OR (Recommendation) OR (Consensus) OR (Opinion)) | 30 |
| Embase | (‘Omicron’ OR ‘XBB.1.5’ OR ‘BQ.1.1’ OR ‘BQ.1.’ OR ‘BA.5’ OR ‘CH1.1’ OR ‘XBB’ OR ‘B.1.617.2’ OR ‘BA.4’ OR ‘B.1.1.529’ OR ‘BA.2’ OR ‘BA.2.12.1’ OR ‘BA.2.75’ OR ‘BN.1’ OR ‘XBB.1.9.1’ OR ‘BA.4.6’ OR ‘BA.5.2.6’ OR ‘BF.7’ OR ‘BF.11’ OR ‘XBB.1.5.1’ OR ‘XBB.1.5’ OR ‘BA.2.75.2’ OR ‘BN1’) AND (‘Guideline’ OR ‘Practice guideline/de’ OR ‘Expert opinion’ OR ‘Recommendation’ OR ‘Consensus’ OR ‘Opinion’) | 37 |
| Total: 225  Total – all duplicates: 135 | |  |
| Date of search: March 31, 2023 | | |

| Recent Covid variants of concern | | | | Keywords |
| --- | --- | --- | --- | --- |
| XBB.1.5 | Omicron | BN.1 | XBB.1.5.1 | Guideline |
| BQ.1.1 | B.1.617.2 | BA.4 | XBB.1.9.1 | Expert opinion |
| BQ.1. | B.1.1.529 | BA.4.6 | XBB.1.5 | Recommendation |
| BA.5 | BA.2 | BA.5.2.6 | BA.2.75.2 | Consensus |
| CH1.1 | BA.2.12.1 | BF.7 | BN1 | Opinion |
| XBB | BA.2.75 | BF.11 |  |  |
